# Residues Clustered in the Light-Sensing Knot of Phytochrome B are Necessary for Conformer-Specific Binding to Signaling Partner PIF3

**DOI:** 10.1371/journal.pgen.1000352

**Published:** 2009-01-23

**Authors:** Elise A. Kikis, Yoshito Oka, Matthew E. Hudson, Akira Nagatani, Peter H. Quail

**Affiliations:** 1 Department of Plant and Microbial Biology, University of California Berkeley, Berkeley, California, United States of America; 2 USDA/ARS – Plant Gene Expression Center, Albany, California, United States of America; 3 Department of Crop Sciences, University of Illinois, Urbana, Illinois, United States of America; 4 Department of Biology, Graduate School of Science, Kyoto University, Kyoto, Japan; The University of North Carolina at Chapel Hill, United States of America

## Abstract

The bHLH transcription factor, PHYTOCHROME INTERACTING FACTOR 3 (PIF3), interacts specifically with the photoactivated, Pfr, form of *Arabidopsis* phytochrome B (phyB). This interaction induces PIF3 phosphorylation and degradation in vivo and modulates phyB-mediated seedling deetiolation in response to red light. To identify missense mutations in the phyB N-terminal domain that disrupt this interaction, we developed a yeast reverse-hybrid screen. Fifteen individual mutations identified in this screen, or in previous genetic screens for *Arabidopsis* mutants showing reduced sensitivity to red light, were shown to also disrupt light-induced binding of phyB to PIF3 in in vitro co-immunoprecipitation assays. These phyB missense mutants fall into two general classes: Class I (eleven mutants) containing those defective in light signal perception, due to aberrant chromophore attachment or photoconversion, and Class II (four mutants) containing those normal in signal perception, but defective in the capacity to transduce this signal to PIF3. By generating a homology model for the three-dimensional structure of the *Arabidopsis* phyB chromophore-binding region, based on the crystal structure of *Deinococcus radiodurans* phytochrome, we predict that three of the four Class II mutated phyB residues are solvent exposed in a cleft between the presumptive PAS and GAF domains. This deduction suggests that these residues could be directly required for the physical interaction of phyB with PIF3. Because these three residues are also necessary for phyB-imposed inhibition of hypocotyl elongation in response to red light, they are functionally necessary for signal transfer from photoactivated phyB, not only to PIF3 and other related bHLH transcription factors tested here, but also to other downstream signaling components involved in regulating seedling deetiolation.

## Author Summary

Plants monitor their environment for informational light signals that are used to direct adaptive morphogenic responses. The phytochrome (phy) family of photoreceptors are central to this process. Upon photoperception, phy molecules rapidly translocate to the nucleus where they interact with basic helix-loop-helix transcription factors, termed PIFs (phy-Interacting Factors), and induce gene-expression changes that control morphogenic responses. The molecular determinants in the phy protein responsible for direct intermolecular signal transfer from the activated photoreceptor to transduction partners are undefined. Using random mutagenesis of *Arabidopsis* phyB, coupled with a reverse-hybrid protein-interaction screen, we identified missense mutations in the N-terminal domain that abrogate the binding of the photoreceptor molecule to PIF3. A subset of these mutated phyB molecules retain the capacity for light-signal perception but are defective in the capacity to transduce that signal to PIF3 and other related PIFs. The mutated residues in these molecules are predicted to cluster at the surface of the protein in a structure termed the “light-sensing knot.” These residues are necessary for phyB-regulated growth in the living plant, establishing that the protein region identified appears to function as a component of the molecular interface responsible for direct signal transfer to transduction partners in the cell.

## Introduction

As sessile photoautotrophic organisms, plants live their lives entirely at the mercy of their environment. Therefore, they have evolved the ability to detect even subtle changes in environmental conditions, and to adjust their developmental programs accordingly, to optimize growth, survival and reproduction. Because plants depend on sunlight for energy to drive photosynthesis, they are particularly adapted to detect and respond to changes in light conditions. To this end, plants have three types of sensory photoreceptors, the blue light sensing cryptochromes and phototropins, and the red/far red light sensing phytochromes [1]–[4].

There are five phytochromes in *Arabidopsis thaliana,* designated phyA-phyE [5],[6]. The phytochromes are photoreversible molecular switches, that undergo rapid interconversion between inactive, Pr (for red-light (R)-absorbing) and active, Pfr (for far-red-light (FR)-absorbing) conformations upon sequential absorption of photons of the appropriate wavelength [7]. Upon photoconversion to the Pfr form, the phytochromes undergo translocation to the nucleus where they initiate developmental programs characteristic of growth in the light, resulting in, among other things, an inhibition of hypocotyl elongation, stimulation of cotyledon expansion and greening. phyA is a light labile phytochrome, highly abundant in the dark, but quickly degraded following photoconversion. This phy is responsible for seedling deetiolation in response to continuous far-red light, and for events occurring very rapidly upon initial exposure to red light. The Type II phytochromes, phyB-phyE, are more stable than phyA in light-grown plants and seem to play more prominent roles under longer-term red-light irradiation conditions, with phyB being the predominant photoreceptor under these conditions [6],[8].

phyA and phyB have been shown to physically interact with the basic helix-loop-helix (bHLH) transcription factor PIF3 [9],[10], as well as a number of other PIF3-related bHLHs, PIF1, PIF4, PIF5, PIF6 and PIF7 [11]–[15]. Following photoconversion and nuclear translocation, the interaction of the phytochromes with these factors and nuclear body formation are thought to be the earliest events in phytochrome signaling. PIF3 is necessary for the light-induced regulation of a subset of rapidly light-responsive genes, and plays an important role in greening during seedling establishment [16]. Furthermore, PIF3 is phosphorylated in a manner dependent on interaction with phytochromes in red light, and this phospohorylation precedes PIF3 degradation via the ubiquitin proteasome system [17],[18]. It appears that phy-mediated proteasomal degradation of PIF3 is crucial for the proper timing of expression of light-induced gene expression [19]. Similarly rapid phy-induced phosphorylation and degradation have been reported recently for PIF1 [20],[21], PIF4 [22] and PIF5 [22]–[24].

Due to the importance of the PIF3-phytochrome interaction to early events in photomorphogenic development, studies have been carried out to molecularly dissect the phyA and phyB binding sites on PIF3. The binding site for phyB, termed the Active PhyB (APB) binding site, is located near the N-terminus of PIF3 and was initially identified by sequence similarity of this region of PIF3 to other bHLHs that bind phyB [13]. phyA binds downstream of phyB on PIF3 at a region termed the Active PhyA binding site (APA) [17]. Similarly separate binding sites for phyA and phyB on PIF1 have also been recently reported [21].

The region of the phytochromes to which the phytochrome interacting bHLHs bind is poorly defined, and the N-terminal residues of phyB that are required for the interaction are unknown. The phytochromes consist of an N-terminal photosensory domain and a C-terminal dimerization domain [5],[7],[25]. The N-terminal domain has four subdomains: an N-terminal extension found only in higher plants, a Per/Ant/Sim (PAS)-like domain (PAS), a cGMP phosphodiesterase/adenyl cyclase/FhlA (GAF) domain, and a phytochrome (PHY) domain. Although PIF3 was originally identified by its ability to bind the C-terminal domain of phyB in a yeast 2-hybrid screen [9], it was later shown that PIF3 photoreversibly binds more strongly to the N-terminal phyB domain in vitro, albeit with somewhat reduced affinity compared to binding to full-length phyB [10],[26].

A previous reverse genetic study aimed at identifying regions of phyB required for its signaling activity in vivo, examined deletion derivatives transgenically expressed in *Arabidopsis* for their biological activity. *In planta* analyses of these derivatives showed strikingly that the C-terminal domain of phyB is not required for phytochrome activity *per se*, but is required for dimerization and possibly to attenuate phyB-signaling activity [27]. These findings, taken together with the abovementioned binding studies [10],[26], provide compelling evidence that PIF3, and other phytochrome signaling partners, bind the phyB N-terminal domain.

Missense mutations in phyB have also been identified for the purpose of defining regions of the photoreceptor which are required for particular aspects of phytochrome signaling. Several such mutations, identified in screens for mutants with long hypocotyls, are located in the N-terminal domain. Specifically, Krall and Reed [28] identified G118R, S134G and I208T, and Reed and colleagues [29] identified H283T. In a screen for phyB mutants deficient in nuclear speckle formation, Chen *et. al.* [30] identified point mutations in the phyB N-terminal domain (C327Y, A372T, and A587T). Furthermore, Kretsch *et al.* [31] identified a phyB point mutation (G564E) that is able to adopt the Pfr conformation, but failed to revert back to the Pr form in the dark (reduced dark reversion) resulting in a hypersensitive phenotype in the light. Interestingly, Oka et al. [32] later found that a different residue substitution at this position, G564A, showed faster dark reversion. However, overall, the spectral characteristics were examined for only three of these mutations by these or other authors [31]–[33] leaving open the question of whether signal perception or signal transfer were affected.

More recently, Oka *et al.* [34] performed a genetic screen with a previously characterized transgenic *Arabidopsis* line expressing a transgene-encoded, phyB-N-terminal-domain fusion-protein, designated N651G-GUS-NLS [27]. This protein consists of the N-terminal 651 amino acids of phyB translationally fused in series to green fluorescent protein (GFP), b-glucuronidase (GUS) (which promotes dimerization), and a nuclear localization signal (NLS) (to ensure proper subcellular localization), and is expressed in a *phyB* null mutant background. The transgenic line was mutagenized with ethyl methyl sulfonate (EMS) and screened for a tall hypocotyl phenotype when grown in dim red light. The rationale for this screen, aimed specifically at the identification of mutations in the phyB N-terminal domain, was that this domain alone is sufficient for phyB signaling, provided that it is capable of nuclear translocation and dimerization, as shown earlier by Matsushita et al. [27]. Oka and colleagues [34] reported the identification of 14 novel phyB missense mutations that resulted in long hypocotyl phenotypes, bringing the total such mutants to 22 when combined with the 8 previously identified, as mentioned above [28]–[30],[32],[35]. Oka et al. [34] examined all 22 mutant phyBs and showed that most were disrupted in their ability to undergo normal photoconversion. Of the remainder exhibiting normal spectral properties, four (R110Q, G111D, G112D, and R352K) were of particular interest because they localized to the “light-sensing” knot of the recently solved crystal structure of *Deinococcus radiodurans* bacteriophytochrome [36].

Here, to identify phyB mutants which are defective in binding to PIF3, we performed a yeast reverse-hybrid screen designed to recover phyB missense mutations which abrogate light-induced interaction of the N-terminal domain of the photoreceptor with PIF3. Such mutants were then examined for loss of normal spectral activity, indicative of loss of signal perception capability, and for loss of their ability to physically interact with PIF3 and other bHLH transcription factors, suggestive of the loss of their ability to transduce the light signal. Functional importance to phyB signaling in vivo was assessed for the spectrally active phyB mutants, by evaluating the capacity of the mutant molecule to inhibit *Arabidopsis* hypocotyl elongation in response to continuous red light (Rc). Conversely, we examined the previously identified phyB missense mutations of Oka et al. [34], shown to lack normal signaling activity in vivo by hypocotyl assays, but to retain normal spectral activity, for their ability to bind PIF3 in vitro.

## Results

### Yeast Reverse Hybrid Screen Uncovers phyB N-Terminal Missense Mutations that Are Affected in Binding to PIF3

We developed a yeast reverse-hybrid screen that allowed the identification of the desired missense mutations in the N-terminal domain of phyB. This screen was based on a previously developed modification of the yeast two-hybrid system in which we showed that the phyB N-terminal domain (phyBNT) fused to the Gal4 DNA binding domain of yeast (DBD) interacts photoreversibly with PIF3 fused to the Gal4 activation domain (GAD) in transformed yeast cells [26]. Yeast reverse hybrid screens have been used extensively to identify point mutations that abolish the interaction of two normally interacting proteins. Such screens are based on a negative selection where protein-protein interaction in a yeast 2-hybrid context results in cell death [37]–[42]. Most yeast reverse-hybrid screens reported to date have sought to dissect interactions between yeast or mammalian proteins. To our knowledge, this study represents the first report of a yeast reverse-hybrid screen performed on plant proteins. In principle, the mutations isolated in this type of screen may either affect amino acid residues that are directly involved in the physical interaction of the target protein and its binding partner, or may result in localized structural changes that consequently indirectly interfere with binding. In addition here, because only the Pfr form of phyB is able to bind PIF3, this screen provides the potential to identify mutations that disrupt photoconversion.

**Figure 1:**
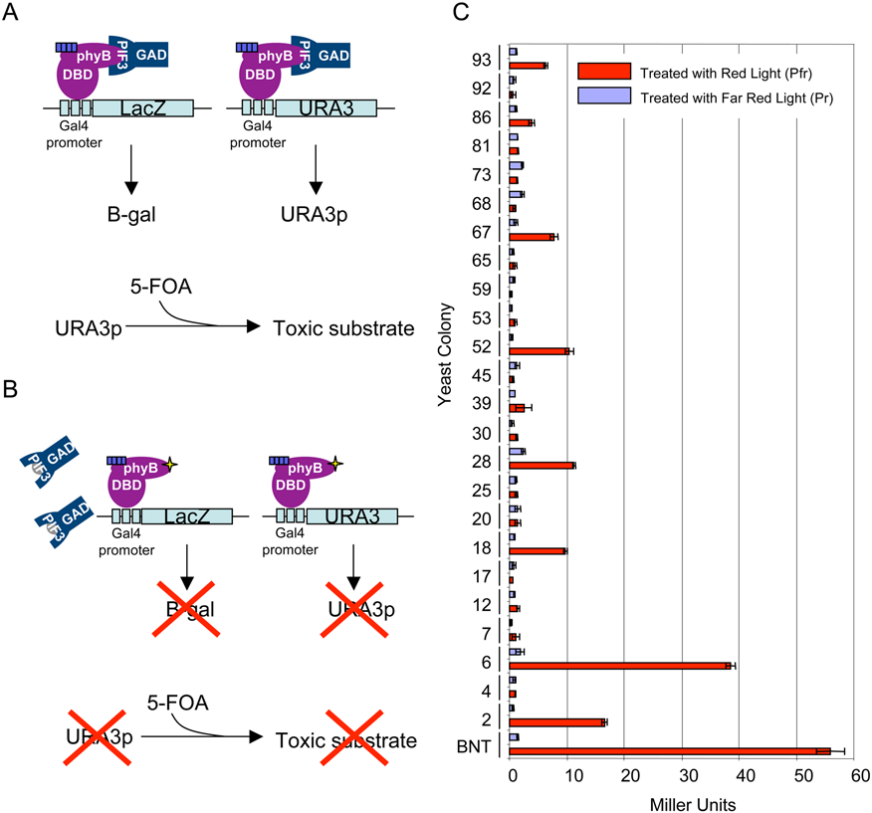
Yeast Reverse-Hybrid Screen Identifies phyBNT Missense Mutations that Result in Reduced Interaction with PIF3. The yeast strain MaV103a, which possesses the genes LacZ and URA3 under the control of Gal4, was transformed with GAD-PIF3- and phyBNT-DBD-containing plasmids or PCR product. A) Schematic showing that in the event of normal, light-induced interaction between phyBNT-DBD and GAD-PIF3 proteins, B-galactosidase and URA3 protein are produced, resulting in cell death on media containing 5-FOA. B) Schematic showing that in the event of a mutation in phyBNT that abolishes interaction with PIF3, little URA3 protein accumulates, resulting in survival even in the presence of 5-FOA. The light blue box represents the chromophore, PCB, and the yellow star represents a missense mutation in phyBNT. C) Enzymatic Assays to test for β-galactosidase activity using ONPG as a substrate were performed for each mutant and the phyBNT wild type control exposed to 5-minute saturating pulses of either red or far red light in the presence of PCB. Red bars represent the interaction in red light and grey bars represent the interaction in far red light. β-galactosidase activity is represented in Miller Units. Technical triplicates were performed and mean values were plotted with error bars representing standard error.

Mutations in phyBNT were generated randomly by error-prone PCR and were screened in red light for loss of interaction with PIF3 in yeast on media supplemented with 5-fluoroorotic acid (5-FOA) and the chromophore phycocyanobilin (PCB). In the event of interaction between phyBNT-DBD and GAD-PIF3, transcription at the *LacZ* and *URA3* genomic loci are activated, resulting in the accumulation of β-galactosidase and URA3 protein (URA3p). The accumulation of URA3p results in death in the presence of 5-FOA. However, a mutation in phyBNT that disrupts binding to GAD-PIF3 prevents transcription of *LacZ* and *URA3* resulting in growth even on 5-FOA. With this yeast reverse-hybrid screening technique, we were able to easily and rapidly obtain large numbers of mutations in phyBNT that disrupt binding to PIF3. A schematic representation of the screening technique is shown in Figure 1, where Figure 1A represents the case in which phyB and PIF3 interact, and Figure 1B represents the case in which a mutation in phyB disrupts binding to PIF3.

Several hundred yeast colonies, co-transformed with mutated phyBNT-DBD and GAD-PIF3, were obtained in the presence of 5-FOA and the chromophore, PCB, in red light, indicative of loss of phyB-binding to PIF3. However, growth on 5-FOA/PCB-containing media alone is an insufficient assay to eliminate mutations that result in the introduction of a premature stop codon in the phyBNT coding sequence, as these would be expected to result in a lack of reporter gene expression during screening. To eliminate this type of false positive from further analysis, immunoblots were performed on crude protein extracts of ninety-five putative positive colonies using an anti-DBD antibody for detection of the transgene-encoded protein. As shown in Figure S1, 35 yeast colonies were identified that accumulated full-length phyBNT-DBD fusion protein. For the remaining colonies examined, no protein was detected with the anti-DBD antibody, presumably due to a stop codon being introduced upstream of DBD in the phyB coding region. The presence of a stop codon in clone #50, which fails to accumulate full length phyBNT-DBD, was confirmed by sequencing.

It is expected that any mutation isolated here that disrupts phyBNT binding to PIF3 would fall within the phyBNT coding region, for the simple reason that this was the only region subjected to mutagenesis. However, the possibility of spontaneously arising second-site mutations resulting in decreased binding affinity or decreased reporter gene expression needed to be ruled out. To this end, plasmid was isolated from each positive yeast colony, recycled through *E. coli*, and re-transformed into the progenitor yeast strain carrying a GAD-PIF3 plasmid. The level of interaction between phyBNT-DBD and GAD-PIF3 in response to saturating 5-minute pulses of either red or far red light was quantified as a function of β-galactosidase activity in liquid assays using ortho-Nitrophenyl-β-galactoside (ONPG) as a substrate. As shown in Figure 1C, all of the yeast plasmids tested exhibited either reduced or completely abolished phyBNT-PIF3 interaction. Specifically, while essentially no interaction significantly greater than the baseline was detected when yeast cells were treated with far red light, 70% of the wild-type level of phyBNT Pfr interaction with PIF3 was detected in clone #6 in response to red light. Clone #s 18, 28, 52, 67, and 93 had approximately 15% of residual binding, whereas clone # 2 had ∼25% of residual binding. The remaining 17 clones tested for interaction with PIF3 using β-galactosidase activity assays, showed no binding in response to red light significantly higher than was detected with the negative control, the Pr form of the wild-type phyB N-terminus (Figure 1C).

### PhyB N-Terminal Mutations in the Context of the Full-Length phyB Protein Disrupt Binding to PIF3 In Vitro

Yeast plasmids were sequenced to identify the mutations responsible for loss of phyB binding to PIF3 in the 24 clones examined by β-galactosidase assays. In many cases, more than one point mutation was identified in a given clone. To assay the identified mutations in the context of the full-length phyB protein, and to distinguish between multiple mutations, individual point mutations were introduced by site-directed mutagenesis into full-length phyB for in vitro translation with a rabbit reticulocyte lysate transcription and translation system (TNT).

In vitro co-immunoprecipitation assays for 47 phyB missense mutations were performed with radiolabeled GAD-PIF3 as bait and radiolabeled phyB as prey as described previously [13]. Due to the relatively large number of mutations identified in each original yeast plasmid, it was not surprising that many of the phyB missense mutations examined did not affect binding to PIF3, when re-assayed as single amino acid changes in an otherwise wild-type protein.

**Figure 2:**
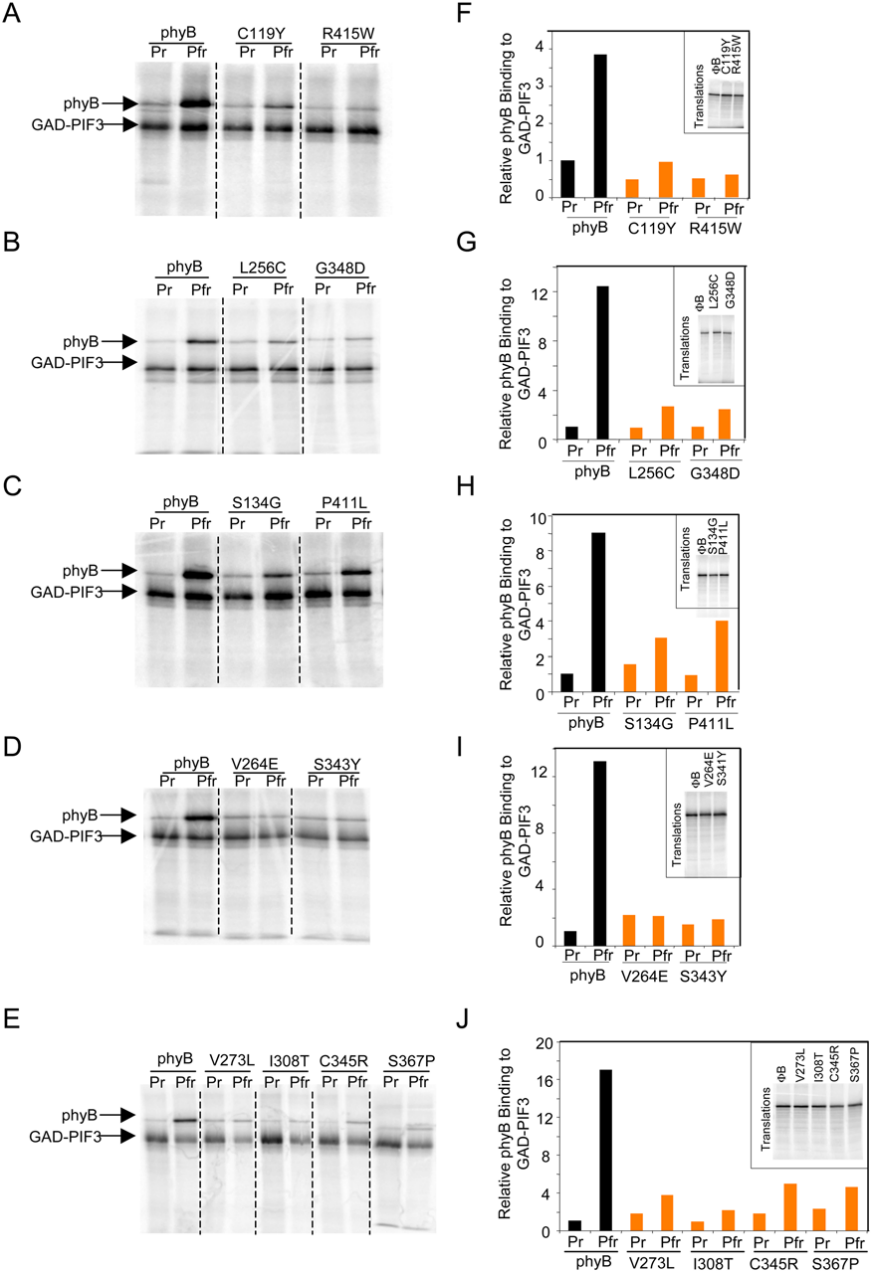
phyB Missense Mutations in the context of the full-length phytochrome Disrupt Binding to GAD-PIF3. PhyB and GAD-PIF3 were synthesized by in vitro translation in the presence of 35S-met. Co-immunoprecipitation assays were performed using GAD-PIF3 as bait and phyB Pr or Pfr as prey. A–E) Co-immunoprecipitations of wild-type phyB compared to phyB missense mutations. The upper band is phyB protein pulled down and the lower band is GAD-PIF3 protein as indicated. F–G) Quantification of binding in in vitro co-immunoprecipitation assays. Black bars represent GAD-PIF3 binding to wild-type Pr or Pfr phyB and orange bars represent binding to the mutants. The inset shows the phytochrome inputs. Binding was quantified relative to phyB input and GAD-PIF3. The wild-type phyB Pr interaction was set equal to one.

**Figure 3:**
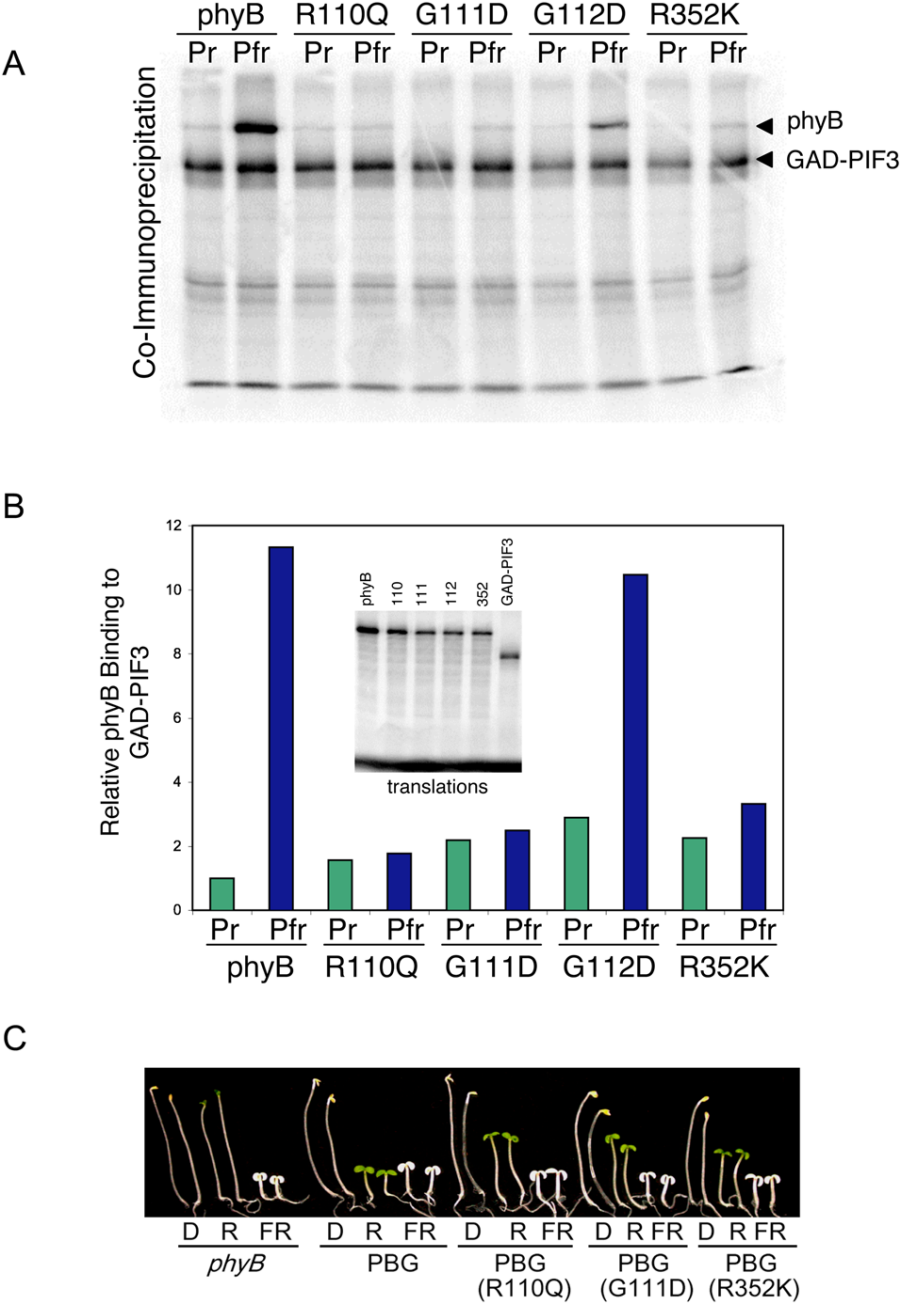
Three phyB Missense Mutants Have Long Hypocotyl Phenotypes and Fail to Bind PIF3 in vitro. A) In vitro co-immunoprecipitation assays of wild-type and mutant Pr and Pfr phyB, performed as in Figure 2. B) Quantification of phyB binding to GAD-PIF3. Black bars represent the wild type interaction and orange bars represent the interaction of the mutants with GAD-PIF3. Inset shows inputs. Quantification is relative to amount of input and amount of GAD-PIF3. Interaction of wild-type Pr phyB with GAD-PIF3 is set equal to one. C) Hypocotyl phenotypes of 4d-old seedlings grown continuously in the dark (D), red (R), or far red (FR) light. Shown are the parental *phyB* null mutant (*phyB*) and transgenic lines expressing either a full-length wild-type phyB-GFP-fusion sequence (PBG) or phyB-mutant variants thereof (R110Q, G111D, R352K) in the *phyB* mutant background.

However, 13 mutations were identified that reduced binding to 50% or less of the wild-type Pfr level in the co-immunoprecipitation assays. Twelve of these are shown in Figure 2. Of these, six (C119Y, R415W, V264E, S343Y, V273L and I308T) displayed undetectable or severely reduced light-induced binding to PIF3, whereas the remainder showed varying levels of limited binding. The thirteenth mutation, G111D, shown in Figure 3A and 3B, also exhibited essentially complete loss of light-induced PIF3 binding. This mutant was of particular interest because, coincidently, it had also been independently identified as one of four in vivo-signaling mutants in the genetic screen for functionally compromised *phyB Arabidopsis* mutants by Oka et al. [34], thereby providing a convergence point for the two studies based on complementary strategies.

To determine whether the three other mutants of Oka et al. [34] (R110Q, G112D and R352K) were affected in PIF3 binding, we generated full-length phyB constructs containing these missense mutations and tested them by co-immunoprecipitation assay. The data show that both R110Q and R352K, like G111D, displayed little or no light-induced PIF3 binding, whereas G112D appears to have been only marginally affected in this capacity by the mutation (Figure 3A and 3B). The seedling deetiolation phenotypes of the three PIF3-binding-deficient mutants generated by Oka et al. [34] are shown in Figure 3C. Each of these mutants displays reduced sensitivity to prolonged continuous R, but responds normally to continuous FR, as demonstrated by Oka et al. [34]. These data indicate that this subset of mutant phyB molecules, disrupted in their capacity to bind PIF3, are also compromised in their capacity to inhibit hypocotyl elongation in response to R light signals. Conversely, the absence of a strong effect of the G112D mutation on PIF3 binding is also consistent with the data of Oka et al. [34] where this mutation was found to have only a weak effect on R-induced hypocotyl inhibition.

### phyB Missense Mutations Fall into Two Distinct Categories

**Figure 4:**
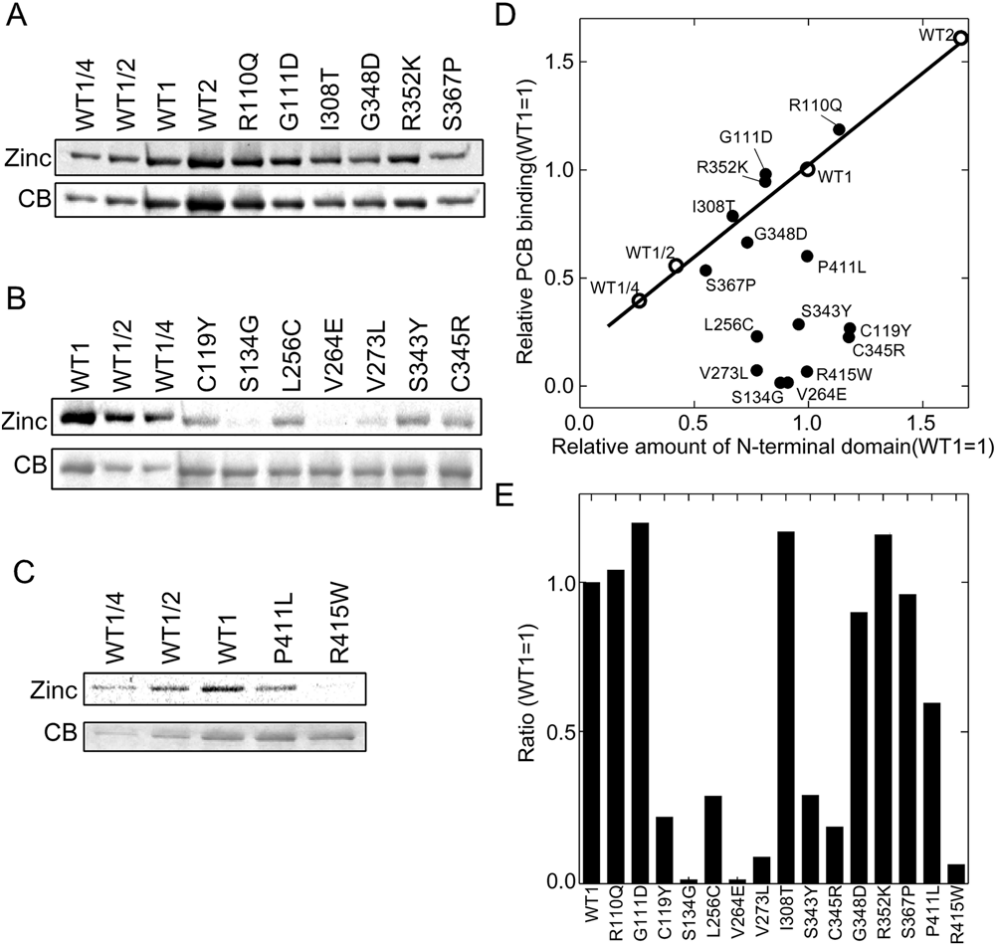
PhyB missense mutants display differential chromophore binding capability. A–C) Representative zinc blots (zinc) for chromophore attachment and corresponding coomassie-blue (CB) staining controls for protein level of recombinant phyBNT (WT) and missense-mutant phyBNT-derivative proteins synthesized in E. coli. D) Scatter plot of chromophore binding of each phyBNT mutant relative to the corresponding amount of recombinant protein for that mutant. Solid line represents the standard curve for wild-type chromophore binding as determined for a dilution series of wild-type phyBNT protein. Open circles represent chromophore binding for wild-type phyBNT with relative amounts of wild-type protein indicated. Closed circles represent chromophore binding for missense mutants. E) Quantification of chromophore binding relative to undiluted (WT1) wild-type phyBNT recombinant protein.

Because wild-type phyB in its inactive, red-light-absorbing, Pr form cannot bind GAD-PIF3 in vitro, one explanation for loss of binding of phyB missense mutants to GAD-PIF3 could be that the mutations disrupt normal phyB photoconversion, thereby preventing the establishment of the Pfr form following irradiation with red light. To test whether the phyB missense mutations identified here are able bind the chromophore, PCB, we performed zinc blot assays, and included the additional two mutants of Oka et al. [34], R110K and R352K, for comparison. Zinc blot assays are based on the fluorescence displayed by bilin-linked polypeptides when they are complexed with zinc ions. As shown in Figure 4, nine phyB mutants (indicated in Table 1) are disrupted in their ability to bind chromophore, despite the chromophore attachment site (C357) being intact. Disruption in this case is defined arbitrarily as a 25% or greater reduction in fluorescence relative to wild-type protein in the zinc-blot assay, although most mutations tested disrupted chromophore binding by greater than 75% (Figure 4E). We refer from here on to these mutants, deficient in chromophore binding, as Class I mutants. Six other mutants (R110Q, G111D, I308T, G348D, R352K, and S367P) were largely unaffected in chromophore binding, as shown quantitatively in a scatter plot (Figure 4D) and bar graph (Figure 4E) relative to wild-type phyBNT. This result is consistent with the previous finding that R110Q, G111D, and R352K all bind PCB [34].

**Table 1:** phyB missense mutations fall into two functional categories.

Amino Acid Substitution	Chromophore Ligation	Photoreversibility	Mutation Position	Mutant Class
C119Y	Reduced	−	PAS	I
S134G	−	−	PAS	I
L256C	Reduced	−	GAF	I
V264E	−	−	GAF	I
V273L	−	−	GAF	I
I308T^1^	+	Aberrant	GAF	I
S343Y	Reduced	−	Knot	I
C345R	Reduced	−	Knot	I
S367P	+	−	GAF	I
P411L	Reduced	−	GAF	I
R415W	−	−	GAF	I
R110Q	+	+	Knot	II
G111D	+	+	Knot	II
G348D	+	+	Knot	II
R352K	+	+	Knot	II

Residues are classified according to the impact the substitution has on the capacity of the phyB molecule to covalently attach the chromophore and undergo normal photoreversible interconversion between the Pr and Pfr conformers. Class I: Fails to ligate chromophore and/or fails to undergo normal photoconversion. Class II: Undergoes normal photoconversion, but fails to bind PIF3. (+) = normal activity. (−) = absence of normal activity. The location of each residue within the three dimensional structure is indicated.

1 Undergoes photoconversion, but Pfr form is spectrally aberrant (see Figure S2).

**Figure 5:**
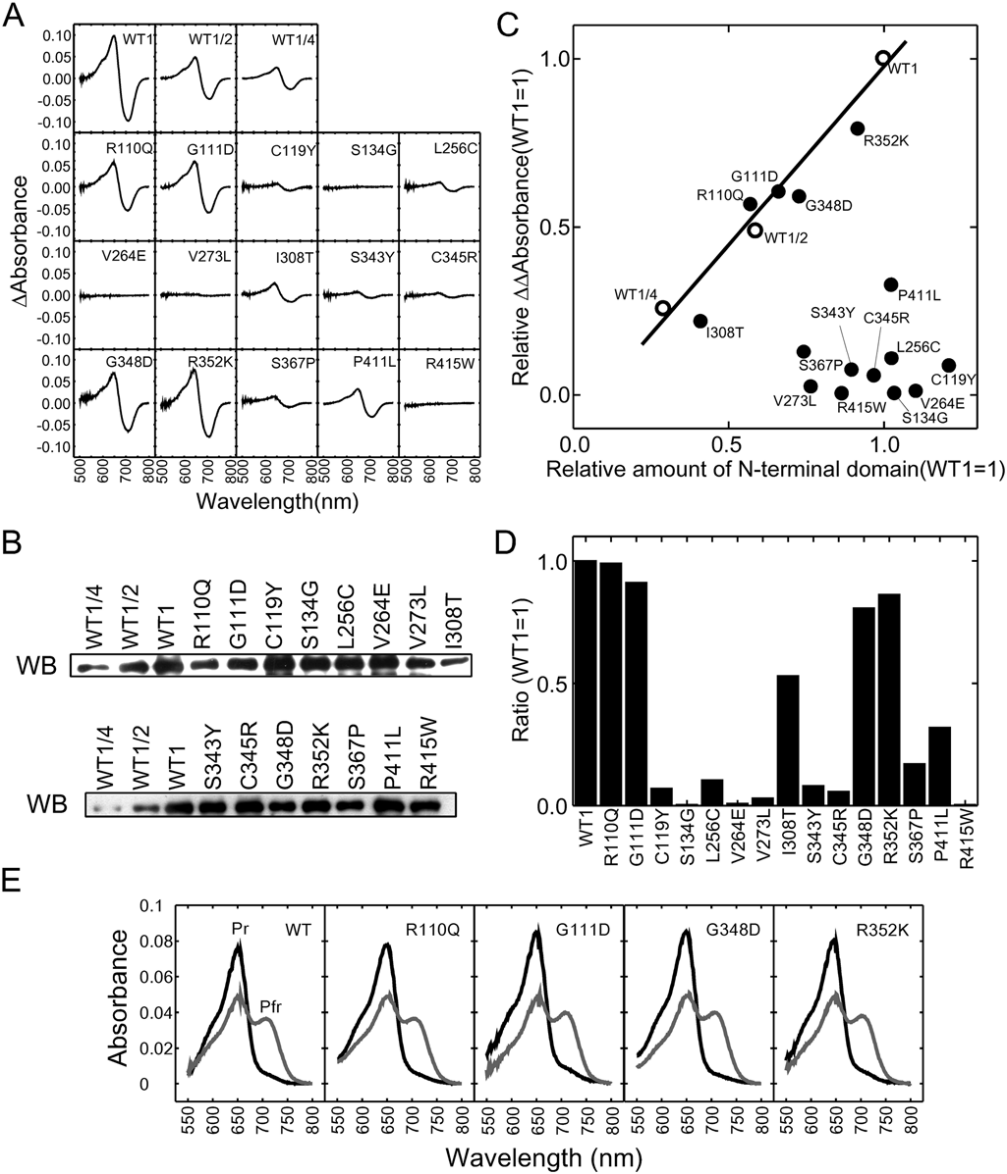
PhyB missense mutants display differential spectral activity. A) Phytochrome difference spectra of recombinant phyBNT (WT) and missense-mutant phyBNT-derivative proteins synthesized in *E. coli.* B) Western-blot (WB) staining of wild-type phyBNT and missense mutants showing relative protein amount. C) Scatter plot showing ΔΔAbsorbance of each missense mutant and wild-type phyBNT normalized to relative protein amount determined from WB staining as in panel B. Solid line represents the wild-type standard curve determined from a dilution series, as in Figure 4D. Open circles represent values for wild-type protein and closed circles represent values for mutant variants. D) Quantification of difference spectra for each mutant relative to wild-type phyB protein. E) Pr and Pfr absorbance spectra for wild-type and Class II phyBNT mutant proteins showing normal spectral properties.

Because zinc blots simply assess chromophore binding, not phytochrome photoreversibility, we measured the Pr-Pfr difference spectra of recombinant phyBNT for each missense mutant (Figure 5). As expected, the mutants that were negative for chromophore binding yielded strongly reduced or no detectable changes in absorbance by difference spectrum analysis, clearly distinct from wild-type (Figure 5A, 5C, and 5D), and consistent with their classification as Class I mutants (Table 1). Strongly reduced in this case is defined arbitrarily as a 50% or greater reduction in absorbance change relative to the wild-type photoreceptor, although most mutations tested disrupted photoconversion by greater than 75% (Figure 5C and 5D). One missense mutant, phyB S367P, which was positive for chromophore binding, failed to show evidence of normal photoreversibility. Similarly, another chromophore-binding-positive mutant, I308T, did display photoreversibility, but the Pfr form was partially bleached and therefore considered to be spectrally aberrant (Figure S2). This behavior is consistent with that reported recently for mutation of the homologous residue (I208) in *Deinococcus* phy [43]. These two phyB mutants, therefore also fall into Class I (Table 1). On the other hand, four mutants, (G348D from the yeast screen, R110Q, and R352K from the previously reported hypocotyl screen, and G111D from both screens) that were positive for chromophore attachment, also showed normal absorbance spectra and photoconversion (Figure 5A, 5C, 5D, and 5E), and are therefore defined as Class II mutants, as indicated in Table 1.

**Figure 6:**
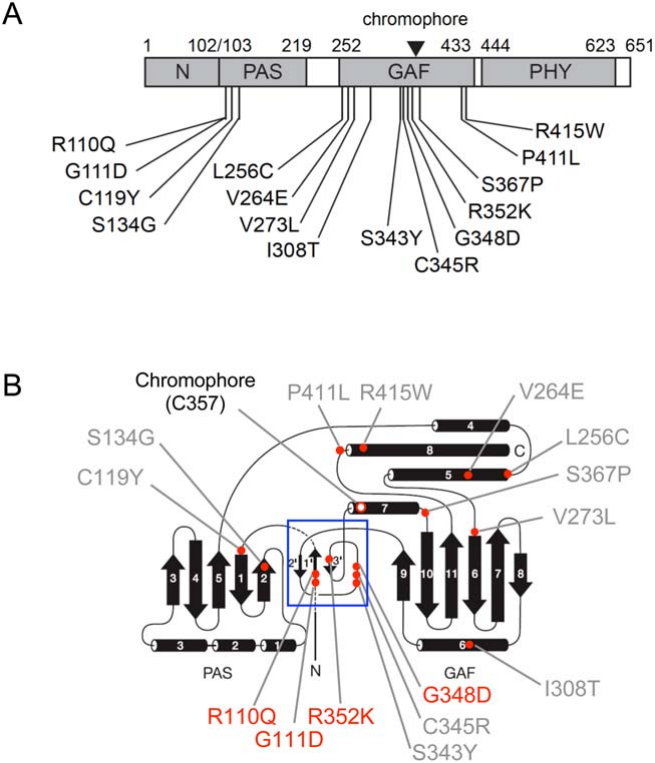
Locations of mutant residues within the N-terminal domain of the phyB polypeptide. A) Schematic of the N-terminal domain of *Arabidopsis* phyB (residues 1 to 651) showing the PAS (residues 103 to 219), GAF (residues 252 to 433), PHY (residues 444 to 623) and N-terminal extension (N) (residues 1 to 102) subdomains, as defined by sequence homology [34], and the locations of the missense mutations investigated here. Inverted triangle indicates chromophore attachment site (C357). B) Schematic representation of *Deinococcus* phytochrome structure adapted from Wagner *et al.* [36]. Black arrows: β-strands. Black cylinders: α-helices. Grey labeling: positions of class I *Arabidopsis* phyB missense mutations. Red labeling – positions of class II *Arabidopsis* phyB missense mutations. Chromophore attachment site (C357) is indicated. Positions of mutated residues are indicated by red dots and were determined from the pairwise alignment of phyB with the *Deinococcus* phytochrome amino acid sequence. Blue box: “light-sensing knot”.

The locations of all 15 mutations within the N-terminal domain of phyB are shown schematically in Figure 6A. The data show that all 15 are confined to the PAS (4 mutations) and GAF (11 mutations) subdomains (Table 1).

### Three phyB Missense Mutations Are Predicted to Reside in a Cleft Formed by the PAS and GAF Domains and May Be Components of the PIF Binding Site

Recently, the chromophore binding domain of phytochrome from the bacterium *Deinococcus radiodurans* was crystallized, and its three dimensional structure solved [36]. This domain is equivalent to the PAS and GAF subdomains of the plant phys (Figure 6A). A pair-wise sequence alignment between *Arabidopsis* phyB and the phytochrome sequence from *Deinococcus* shows that the two proteins have 29% identity over the crystallized region of the *Deinococcus* protein (Figure S3).

Given this sequence similarity between *Arabidopsis* phyB and *Deinococcus* phy, it is possible that their structures are also similar. To predict the location of the phyB missense mutations identified here in the context of a three dimensional structure, we mapped them onto the solved *Deinococcus* structure. The residues corresponding to the missense mutants are indicated on the sequence alignment in Figure S3.

A schematic representation of the three dimensional structure of *Deinococcus* phytochrome, published by Wagner *et al.* [36], is reproduced in Figure 6B showing the locations to which the point mutations identified here map, in addition to the mutant class designation for each highlighted residue. As shown, five Class I mutants (those that fail to bind PCB in zinc blot assays and have abnormal spectral properties) fall in the GAF domain, and two fall in the PAS domain. In addition the Class I mutants, I308T and S367P, which bind PCB but nonetheless have abnormal phyB spectral properties, are also located in the GAF domain, consistent with a function in chromophore-protein interaction. In contrast, all four photoconvertible (Class II) mutants that are affected in PIF3 binding and result in a long hypocotyl phenotype in Rc are located in a trefoil loop, at the junction of the PAS and GAF domains, also referred to previously as the light-sensing knot [36],[43] (Figure 6B; Table 1).

**Figure 7:**
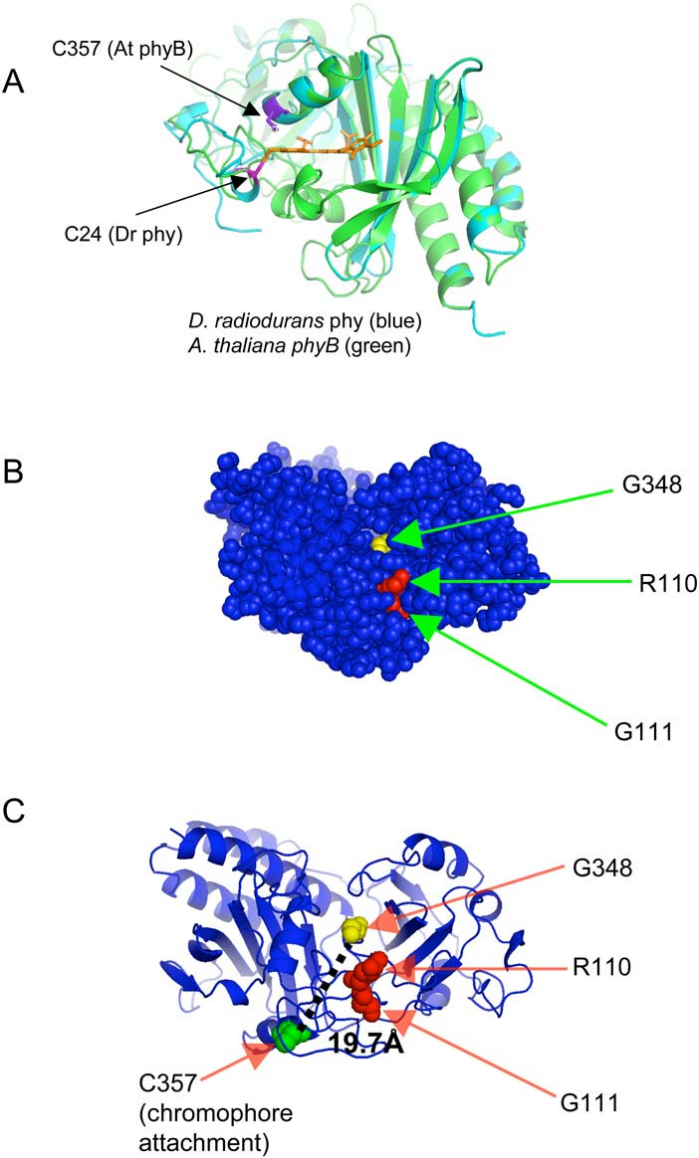
Predicted phyB Structure Based on Homology with Deinococcus phytochrome. The *Arabidopsis* phyB sequence was threaded onto the *Deinococcus* phytochrome. A) Superimposition of ribbon diagrams of the homology model of *Arabidopsis* phyB N terminus and experimentally determined structure of *Deinococcus* phy (pdb: 1ztu). *Deinococcus* is represented in blue and *Arabidopsis* in green. There is complete agreement between the main chain positions of the model and the experimental structure in the chromophore binding region. The cys24 residue of *Deinococcus* phytochrome is indicated in magenta. The chromophore binding cys357 of *Arabidopsis* phyB is indicated in purple, with the sulfur atom highlighted, showing the conserved spatial location of the chromophore binding cysteine. B) Space filling diagram of the predicted phyB structure, with three of the Class II amino acid residues identified in this study shown in yellow (G348) or red (R110, G111), showing that they are at least partially exposed to the surface, and located in close proximity to one another in the cleft at the junction of the PAS and GAF subdomains. C) Ribbon diagram of the predicted phyB structure with the three apparently solvent exposed residues shown in red or yellow space filling format and the chromophore attachment site shown in green space filling format. The distance between G348 and the chromophore attachment site (C357) is indicated by the dashed line.

To gain further insight into the potential locations of the mutated residues within the three dimensional structure of *Arabidopsis* phyB, the PAS-GAF segment of the phyB sequence corresponding to the crystallized chromophore-binding domain of *Deinococcus* was threaded onto the *Deinococcus* crystal structure (pdb: 1ztu). The homology model was produced using the program “nest” [44] which was found to make the fewest mistakes overall in a comparison of available homology modeling programs [45]. The resultant homology model is shown in Figure 7A, with green ribbons indicating the *Deinococcus* crystal structure, and blue ribbons indicating the predicted *Arabidopsis* phyB structure, with the position of the chromophore in *Deinococcus* superimposed in gold. The close agreement between the *Deinococcus* structure and the homology model is consistent with a high level of conservation in the critical structural residues. The sulfhydryl group of the *Arabidopsis* chromophore-binding cysteine residue is co-ordinated with the position of the ethylidene moiety on the chromophore sufficiently closely and in the correct conformation to form the thioether bond by which the chromophore is known to be covalently attached [25]. This is true despite the fact that the cysteine residue is in a very different position in the primary sequence of the protein from that in *Deinococcus*, and that the cysteine residue approaches the chromophore from the opposite side of the plane of the bilin from the side from which it binds in the *Deinococcus* structure (Figure 7A). This is consistent with the predictions of Wagner et al. [46] on the position of the plant chromophore-binding site.

Examination of the predicted phyB structure using the software PyMol revealed that three of the Class II phyB residues in the knot region, R110Q, G111D and G348D, described here as being required for binding to PIF3, may be solvent exposed. These presumptive surface residues appear to be clustered near each other at the interface between the PAS and GAF domains as shown in Figure 7B.

As shown in the alignment in Figure S3, one of these residues, G348D, also appears to be close to the chromophore binding site (C357) in the primary phyB sequence. Figure 7C shows a 3D ribbon diagram of the predicted phyB structure with the three surface residues and the chromophore attachment site shown in space-filling format. The distance between G348 and the chromophore attachment site is predicted to be ∼19.2 Å.

To determine if any of the phyB residues identified here as being required for binding to PIF3 are conserved among *Arabidopsis* phytochromes, a multiple sequence alignment of phyA-phyE was constructed using the Muscle algorithm. As shown in Figure S4, all but four of the 15 residues described here are conserved amongst all five phytochromes. Given the differential affinity of PIF3 for phyB compared to the other phytochromes, especially phyA which has been shown to bind a different region of PIF3 than that to which phyB binds (APA vs. APB) [17] we might predict that the phyB residues directly involved in binding to PIF3 would not be conserved in phyA. Of the four Class II mutations that had normal photoconversion but led to a disruption in phyB binding to PIF3, one residue, R110 is a lysine in phyA. As shown above, the same substitution in phyB results in lack of binding to PIF3, suggesting that this residue may make a significant contribution to the differential affinity of phytochromes for PIF3.

### phyB Mutations that Disrupt Binding to PIF3 Disrupt Binding to all the bHLH PIFs

**Figure 8:**
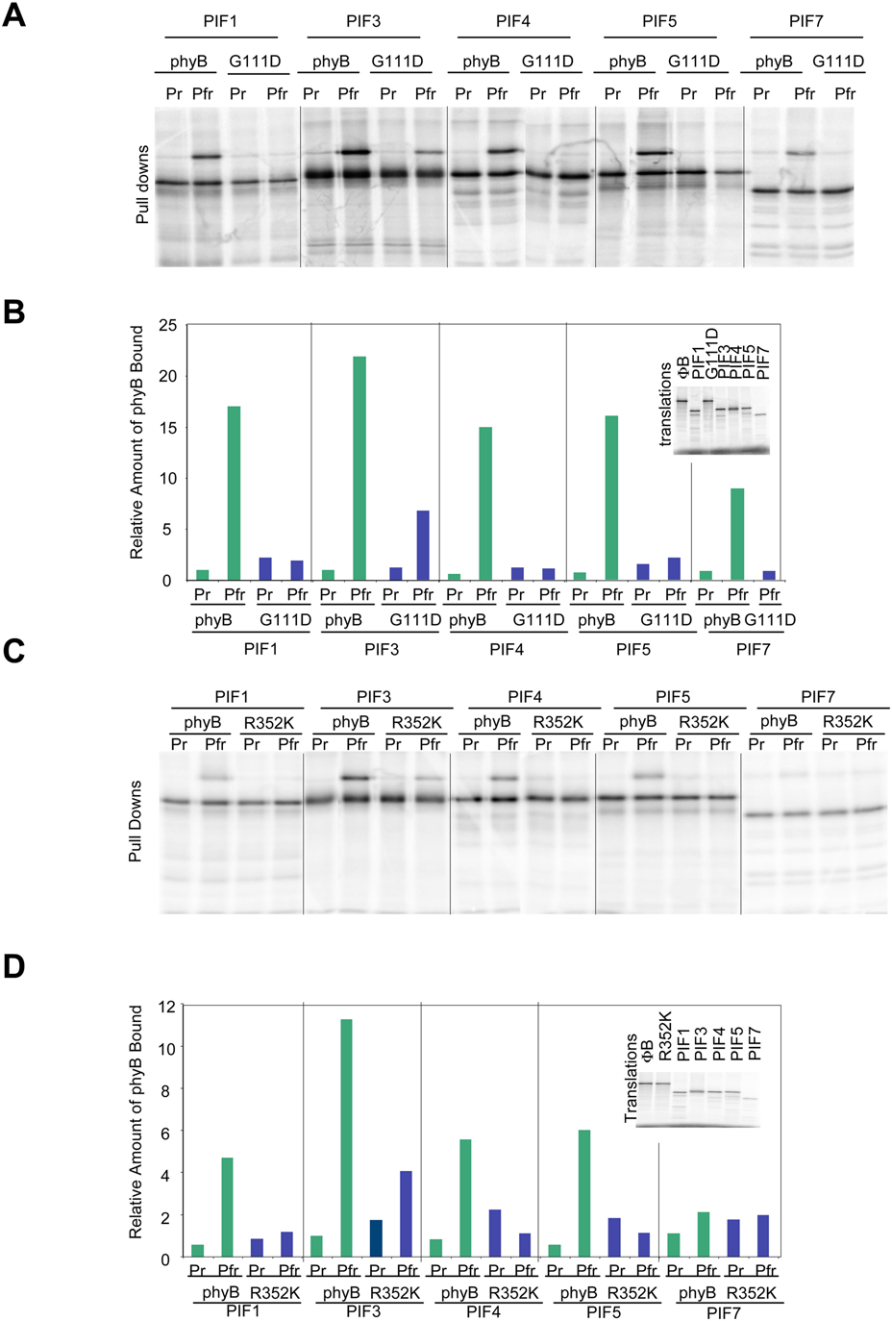
The Two Class II phyB Missense Mutations Tested Abolish Binding to Multiple bHLH PIFs. A) Co-immunoprecipition assays of the full-length phyB G111D mutant compared to wild type phyB for interaction with PIF1, PIF3, PIF4, PIF5, and PIF7. B) Quantification of the interaction of the phyB G111D mutant with the bHLHs shown in panel A. Green bars represent binding of the wild-type phyB and blue bars represent binding of the mutant. Interaction of the wild-type phyB Pr with GAD-PIF3 is set equal to one. C) Co-immunoprecipitation assays of the full-length phyB R352K mutant compared to wild-type phyB for interaction with PIF1, PIF3, PIF4, PIF5, and PIF7. D) Quantification of the interaction of the phyB R352K mutant with the bHLHs shown in panel C. Green bars represent binding of the wild-type phyB and blue bars represent binding of the mutant. Interaction of the wild-type phyB Pr with GAD-PIF3 is set equal to one.

Disruption of the PIF3 binding site on phyB may disrupt binding to all the phyB-interacting bHLHs, because phyB has been shown to bind to the APB domain present in all phy-interacting bHLHs [13]. To test this hypothesis, phyB G111D and R352K, two Class II phyB mutants (those that photoconvert but do not bind PIF3), were tested for binding to PIF1, PIF3, PIF4, PIF5, and PIF7 by in vitro co-immunoprecipitation assays. As shown in Figure 8, these two mutations do indeed disrupt binding to all of the bHLH PIFs tested, and would therefore be predicted to be qualitatively more impaired in red light signaling than plants deficient in individual bHLH PIFs, all of which have overlapping and unique roles in phytochrome signaling [5], [11], [16], [19], [20], [22], [47]–[51].

## Discussion

### phyB-PIF-Interaction Mutants Identify Amino Acids Functionally Critical to the Pfr-Specific Intermolecular Signal-Transfer Reactions that Induce Seedling Deetiolation In Vivo

The data from our yeast-reverse-hybrid screen presented here have identified a set of amino acid substitutions in the N-terminal domain of phyB that disrupt the Pfr-specific interaction with PIF3 and related bHLH transcription factors. A number of phyB missense mutants have been identified previously in genetic screens, but they have not been characterized for their ability to bind signaling partners [28]–[30],[32]. The success of our screening strategy suggests its potential utility in studying other plant signaling systems that depend on protein-protein interactions. Moreover, the remarkable convergence of our molecular screen and the independent phenotypic screen of Oka et al. [34] on at least one critical signaling residue, together with our demonstration that, conversely, two additional long-hypocotyl phyB-signaling mutants from the latter screen also fail to bind PIF3, is compelling evidence that the residues identified by the molecular-interaction assay are functionally relevant to seedling deetiolation.

The phyB mutations identified here fall into two functionally distinct categories: those disrupted in light-signal perception (Class I), because of defective chromophore function, and those normal in signal perception, but defective in the capacity of the Pfr form to bind to PIF3 (Class II). One subset of the Class I mutants are defective in chromophore ligation, and therefore lack the capacity to absorb the light signal, whereas a second subset appear to support normal ligation but display a lack of, or abnormal, photoconversion activity. In either case, the photoreceptor is unable to undergo normal light-activated conversion to the active Pfr conformer necessary for PIF3 interaction. By contrast, the Class II mutants exhibit normal light-induced conversion to the Pfr form, indicating normal photoperception, but the mutant molecule is altered in determinant(s) necessary for the correct physical interaction between the two signaling partners, with the result that signal-transfer to PIF3 is abrogated.

However, because of the well-established observation that simple mutation of PIF3 does not phenocopy mutation of phyB [14],[16],[19],[52], PIF3 alone cannot be responsible for transducing all phyB signaling involved in controlling the multiple, pleiotropic facets of the overall seedling deetiolation process. In fact, the *pif3* single mutant is hypersensitive to Rc, the converse of the extreme hyposensitive phenotype of the *phyB* mutant [14],[16],[19],[52]. Thus, the apparent pleiotropic loss of phyB signaling activity *in planta* by the Class II mutants identified here suggests that these residue substitutions cause a global disruption of the phyB signaling necessary for the overall deetiolation process. This implies in turn, that these mutations disrupt productive signaling interactions with one or more factors in addition to PIF3, and that these factors collectively or alone transduce the signals to multiple downstream pathways necessary for deetiolation.

Other potential candidates for this role include the other known phy-interacting, PIF3-related bHLH transcription factors. Consistent with this possibility, both Class II mutants tested here (G111D and R352K) displayed loss of Pfr-specific binding capacity for several of these factors, PIF1, PIF4, PIF5 and PIF7. However, while this result indicates the broader importance of these residues for phy recognition of this general class of signaling partner, both single and higher-order mutations in these factors, like PIF3, also cause Rc-hypersensitivity [14],[53],[54], or have no effect [12], rather than the hyposensitivity expected for loss of phyB signaling. One possible mechanism explaining this apparent contradiction is suggested by the recent twin observations that PIF1, PIF3, PIF4 and PIF5 act collectively to repress seedling deetiolation [55], and that interruption of phyB-PIF interaction, through PIF mutation, leads to increased levels of the PIF protein [19]. Consequently, the concomitant loss of phyB binding to these multiple PIFs, through phyB mutation, in the present study, would be expected to result in increased levels of these factors in the light [19], without the complication of antagonism imposed by the increased levels of active phyB known to occur under these conditions [14],[19],[54]. These increased levels of the PIFs would then be predicted to repress the normally phyB-induced facets of deetiolation, resulting in hyposensitivity, as observed here for the Class II *phyB* mutant lines (Figure 3C). A second possible alternative mechanism would suggest the potential existence of yet additional components that, at least partially, utilize the same interaction site on phyB as the known PIFs for the primary signal transfer process from the activated photoreceptor necessary for inducing seedling deetiolation.

Regardless of the specific underlying mechanism, this binding site appears to have a very fundamental pleiotropic function in the primary, intermolecular signaling process. The alternative, that the mutations at this site could significantly alter the phyB three-dimensional structure, such that binding of other factors at a distant location is disrupted seems less likely. This is because chromophore binding and photoconversion - processes known to be sensitively dependent on an intact three-dimensional structure [7],[43] - do not seem to be affected by these mutations. Regardless, although the interaction with the PIFs identified here appears unlikely to be responsible for all phyB signaling, these interactions do appear to provide a useful marker for the apparently broader ensemble of signal-transfer interactions collectively responsible for all phyB signaling necessary for inducing normal seedling photomorphogenesis.

### Potential Structure-Function Implications of phyB Mutations

Based on sequence similarities to known protein structural domains, the phy protein has been defined as being composed of a series of subdomains [7],[25], as shown in Figure 6A. The discovery, therefore, that all of the mutations identified here are confined to the PAS and GAF subdomains of the phyB N-terminal domain (Figure 6A) is evidence of the centrally important role played by these domains in the photoreceptor's function. This prompted us further to examine the locations of the presumptive homologous residues by utilizing the crystal structure of the PAS-GAF domains of the bacteriophytochrome from *Deinococcus radiodurans* [36],[43]. This analysis led to intriguing insights into the possible location of the postulated PIF-interaction surface within the three-dimensional structure of the photoreceptor and its functional importance to phyB signaling in the cell.

### Most phyB Missense Mutations Disrupt Photoperception

The majority (eleven) of phyB mutants identified here in the yeast reverse-hybrid screen were found to be Class I (Table 1). Of these, nine have reduced, or lack detectable, chromophore ligation to the apoprotein, suggesting that the affected residues are either directly necessary for the catalytic chromophore-ligase activity intrinsic to the molecule, or indirectly necessary for the structural integrity of the catalytic site. The remaining mutants, I308T and S367P, have normal chromophore ligation but fail to undergo normal photoconversion. Regardless, both subclasses of mutations eliminate or severely reduce the light-induced conversion of the photoreceptor to its active Pfr form, thereby abrogating the photoperception function of the molecule. Nine of these mutations are located either in the GAF domain (seven residues), which contains the chromophore attachment site and surrounding binding pocket [36],[43], considered to be required to stabilize the protein-chromophore interaction [56], or in the adjacent knot region (two residues) of the protein, in the vicinity of the chromophore [36]. It is perhaps not surprising, therefore, that these residues might have an important functional role in maintaining the structural and/or catalytic integrity of the protein interface with the chromophore. Only two Class I residues are in the PAS domain (Table 1).

### The Trefoil Knot Region of phyB Has a Critical Role in Binding to PIF3

In contrast to the Class I mutants, all four Class II mutants (R110Q, G111D, G348D and R352K) are strikingly clustered in the “light-sensing” trefoil knot at the interface between the PAS and GAF domains of the *Deinococcus* phy molecule [36] (Figure 6B). As indicated above, three of these residues (R110Q, G111D, and R352K) were also identified in the screen for long hypocotyl mutants. The fact that all of our Class II mutants appear to be physically clustered, provides compelling additional support for the idea that the trefoil knot region of phyB is required for normal phyB signaling capabilities, as suggested previously [34].

To further examine the potential three-dimensional spatial distribution of the mutant residues described here, a predicted *Arabidopsis* phyB three-dimensional structure was generated based on homology to the *Deinococcus* phytochrome with a solved crystal structure (Figure 7). This analysis revealed that three of the four Class II residues are not only predicted to be clustered near each other, but also to be solvent exposed in a cleft formed by the junction of the PAS and GAF domains (Figure 7). This finding suggests that these residues (R110, G111, and G348) are surface exposed. Although it is possible that other domains of phyB not included in the homology model cover this region in the full protein structure, the role of these residues in interaction with PIF3 suggests that they are exposed in at least the Pfr form of the molecule. They are, therefore, potentially positioned within the photoreceptor molecule where local conformation and/or accessibility may be switchable upon reversible light-induced conversion between the two phyB conformers, thereby providing at least part of the conformer-specific binding site on phyB for interacting signaling partners.

### Conclusions

The data presented here have identified two classes of amino acid residues with functionally distinct roles in the photoregulatory activity of the phyB photoreceptor molecule: Class I residues which are necessary for the correct perception of incoming light signals, and Class II residues which are functionally necessary for the transfer of perceived signals to one or more categories of interacting partners in the intracellular transduction chain controlling light-induced seedling development. The apparent surface location of three of the Class II residues, clustered in the predicted PAS-GAF knot interface region of the phyB molecule, and their necessity for conformer-specific binding to the PIF bHLH transcription factors, suggest that these residues may comprise at least part of the signal-transfer site on the activated photoreceptor.

## Materials and Methods

### Yeast Growth

The yeast strain MaV103a was used for the reverse-hybrid screen. This strain was maintained on YPD plates or on L-W- SD-media for plasmid selection. Media was prepared according to BD Biosciences Clontech (Palo Alto, CA).

### Mutagenesis of the PhyB N-Terminal Domain

The phyB N-terminal domain (phyBNT) corresponding to the first 1863 nt of phyB from the start codon, was mutagenized by PCR with Mutazyme™ (Stratagene, La Jolla, CA). The PCR primers contained phyB sequence flanked by sequence from the D153 (DBD-containing) vector. Primer sequences were as follows:

5′ CAACTCCAAGCTTGGATCCGTCGACCCCGGGGCATGGTTTCCGGAGTCGGGGGTAGTG

5′ GCTTGTTCGATAGAAGACAGTAGCTTCATTTGCGGCCGAGATTCTTTAAAAGAGTCTCTCAG

The underlined portion of primer sequences indicates phyB sequence.

PCR product was purified with a Qiaquick PCR purification Kit (Qiagen, Valencia, CA).

To insert the PCR product into the D153-DBD yeast vector, D153 was digested with NotI and overhangs were filled in by Klenow. GAD-PIF3 carrying Mav103a yeast cells were then transformed with PCR product and linearized D153 vector. Circular plasmid was reconstituted by GAP repair in yeast as described previously [57],[58].

### Yeast Reverse-Hybrid Screening Conditions

MaV103a cells transformed with GAD-PIF3, phyBNT mutagenized PCR product, and linearized D153 vector were grown for 3 d under 1 µmol/m^2^s Rc on L-W- SD plates containing 0.035% 5-FOA and 25 µM PCB. Colonies were then transferred to L-W- SD plates for maintenance, or W- SD plates to lose the GAD-PIF3 plasmid.

### Isolation of Yeast Plasmids

Yeast plasmids were isolated from overnight liquid cultures using the YEASTMAKER™ yeast plasmid isolation kit according to the manufacturer's instructions (Clontech, Palo Alto, CA).

### Yeast Transformation

When large numbers of yeast transformants were required, as in screening, yeast transformation was performed as previously described [59]. When fewer transformants were required, transformation was performed with the Fast™-Yeast Transformation Kit according to the manufacturer's instructions (Genotech, St. Louis, MO).

### β-Galactosidase Quantitative Liquid Assays

β-galactosidase assays were performed as described previously [26], with some modifications in growth condition and light treatments. 1 mL cultures were grown overnight in the dark in L-W- media containing 25 µM PCB. Overnight cultures were divided into two equal parts and treated with saturating pulses of 5 minutes of either red or far red light. 2 mL of YPD media containing 10 µM PCB was added to each culture and cultures were incubated for 2 hrs in the dark. Light treatments were then repeated and cultures returned to the dark for 1 hr. From that point on, assays were performed with technical triplicates as described by Clontech [60].

### Site-Directed Mutagensis

Site-directed mutagenesis of phyB was performed using the Quick Change Site-Directed Mutagenesis kit from Stratagene (La Jolla, CA) using complementary sense and anti-sense oligonucleotides containing the desired mutation.

### Immunoblot Analysis

SDS-polyacrylamide gel electrophoresis, protein blotting, and immunodetection were performed as described [14].

### In Vitro Co-Immunoprecipitation Assays

In vitro co-immunoprecipitation assays were performed as described previously [13].

### *E coli* Expression of phyB, Zinc Blots, and Difference Spectra Analysis

For phyBNT protein expression, the wild type and mutant phyBNT fragment was cloned into the pTYB2 vector containing an Intein/CBD tag (New England Biolabs). *Escherichia coli* transformation and expression of wild type- and mutant phyBNT-Intein/CBD fusion proteins were performed as previously described [32],[34]. For Zn blot assays, the phyBNT-Intein/CBD fusion proteins were affinity-purified with a chitin column according to the manufactuer's instructions (New England Biolabs). Purified phyBNT-Intein/CBD fusion proteins were incubated with PCB at 4°C for one hour and subjected to the assay for chromophore attachment as previously described [32],[34]. For difference spectra analysis, the crude extracts from *Escherichia coli* were directly incubated with PCB at 4°C for one hour. Zn blot and difference spectra analysis were performed as described previously [32],[34].

### Plant Growth and Hypocotyl Measurements

Seeds were sterilized, stratified and induced for germination as described [16], then grown in darkness, Rc or FRc at 21°C for 5 days. Seedlings were pressed gently onto the surface of agar medium before photographs were taken.

### Protein Sequence, Structure, and Homology Modeling

Throughout this work, the phyB sequence of *Arabidopsis* ecotype Columbia (GenBank accession number: NP_179469) was used. The PyMOL package (http://pymol.sourceforge.net/) was used for visualization of protein structures. Homology models were generated using the nest [44] program of the Jackal package. The 1ztu PDB accession of the *Deinococcus* structure [36] was used to generate all structural diagrams and homology models.

## Supporting Information

Figure S1 Western Blot Screening of Yeast Colonies for Full-Length phyBNT-DBD Fusion Protein. Protein was isolated from each yeast colony obtained on screening plates (indicated by number) and was probed with antibody raised against the Gal4 DNA Binding Domain (anti-DBD). The presence of a band indicates that the yeast colony produced full-length fusion protein (compare with unmutated control (BNT)). The absence of a band indicates the introduction of a nonsense mutation resulting in a premature stop codon.
(2.71 MB TIF)

Figure S2 phyB Missense Mutation I308T Causes Aberrant Spectral Properties. Superimposed difference spectra of wild-type and I308T missense mutant of phyB showing reduced absorbance change of the Pfr form compared to the mutant in the far-red region of the spectrum.
(0.58 MB TIF)

Figure S3 *Arabidopsis* phyB Missense Mutations Map Throughout the region of the *Deinococcus radiodurans* Phytochrome Used for Structure Determination. Pairwise sequence alignment between *Arabidopsis* phyB and phytochrome from the bacteria *Deinococcus*. phyB - *Arabidopsis* phyB; Dr - *Deinococcus* phytochrome; E-value - 1.16384E-28; 29% identical. Conserved residues are indicated. Boxes indicate positions of *Arabidopsis* phyB missense mutations that disrupt binding to PIF3. Oval indicates position of chromophore attachment in *Arabidopsis*.
(0.41 MB TIF)

Figure S4 Multiple Sequence Alignment of *Arabidopsis* Phytochromes. Conserved residues are highlighted in reverse contrast. Arrows indicate amino acid residues defined here as being required for phyB binding to PIF3. Red arrows mark conserved residues, and green arrows mark non-conserved residues.
(3.13 MB TIF)
