# Supplementary figures and images for: Residues Clustered in the Light-Sensing Knot of Phytochrome B are Necessary for Conformer-Specific Binding to Signaling Partner PIF3
Source: PLoS Genet. 2009 Jan 23;5(1):e1000352. doi: 10.1371/journal.pgen.1000352 (PMC2621353; doi:10.1371/journal.pgen.1000352)

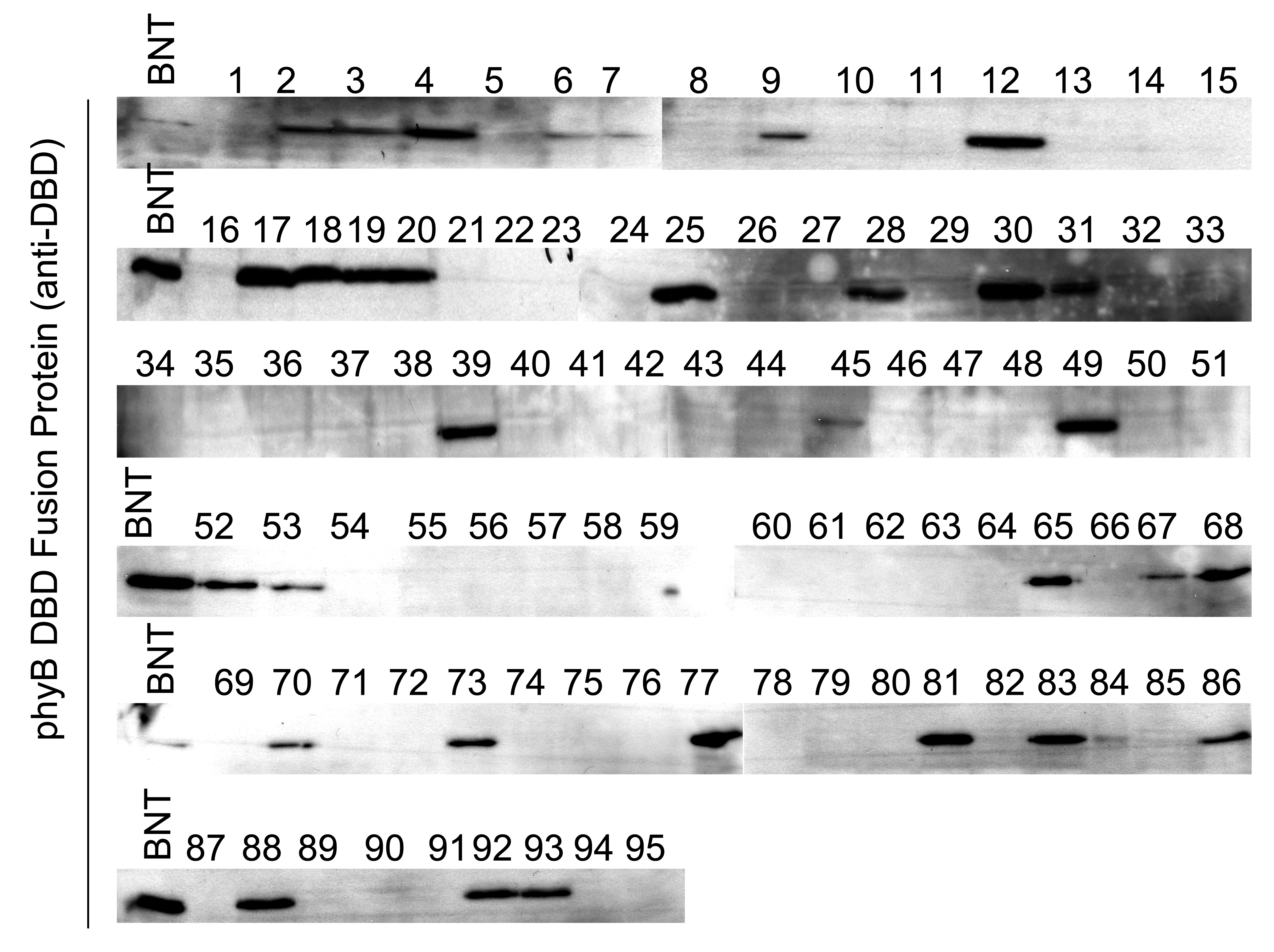

Supplement: Figure S1 — Western Blot Screening of Yeast Colonies for Full-Length phyBNT-DBD Fusion Protein. Protein was isolated from each yeast colony obtained on screening plates (indicated by number) and was probed with antibody raised against the Gal4 DNA Binding Domain (anti-DBD). The presence of a band indicates that the yeast colony produced full-length fusion protein (compare with unmutated control (BNT)). The absence of a band indicates the introduction of a nonsense mutation resulting in a premature stop codon. (2.71 MB TIF) [file pgen.1000352.s001.tif]

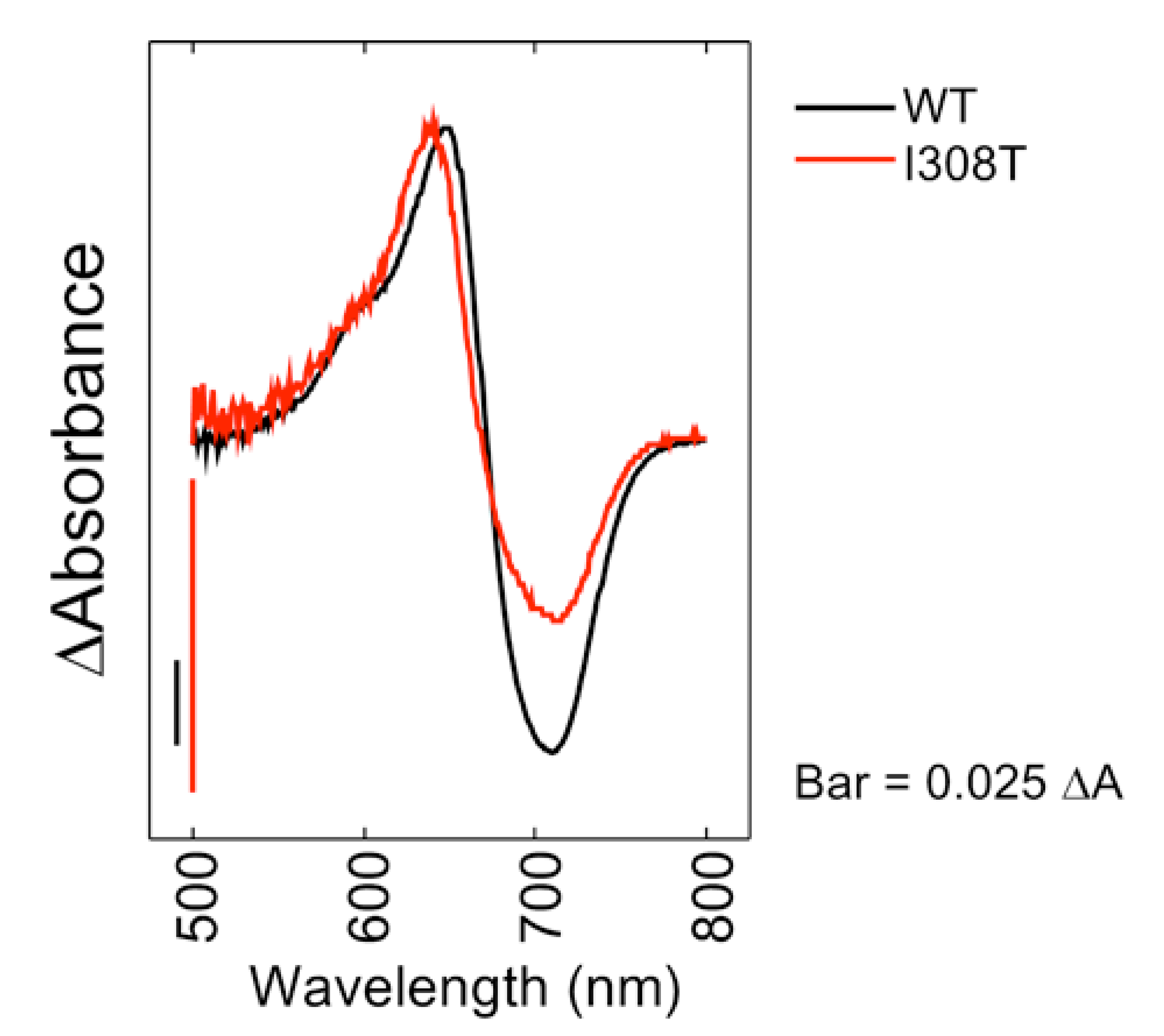

Supplement: Figure S2 — phyB Missense Mutation I308T Causes Aberrant Spectral Properties. Superimposed difference spectra of wild-type and I308T missense mutant of phyB showing reduced absorbance change of the Pfr form compared to the mutant in the far-red region of the spectrum. (0.58 MB TIF) [file pgen.1000352.s002.tif]

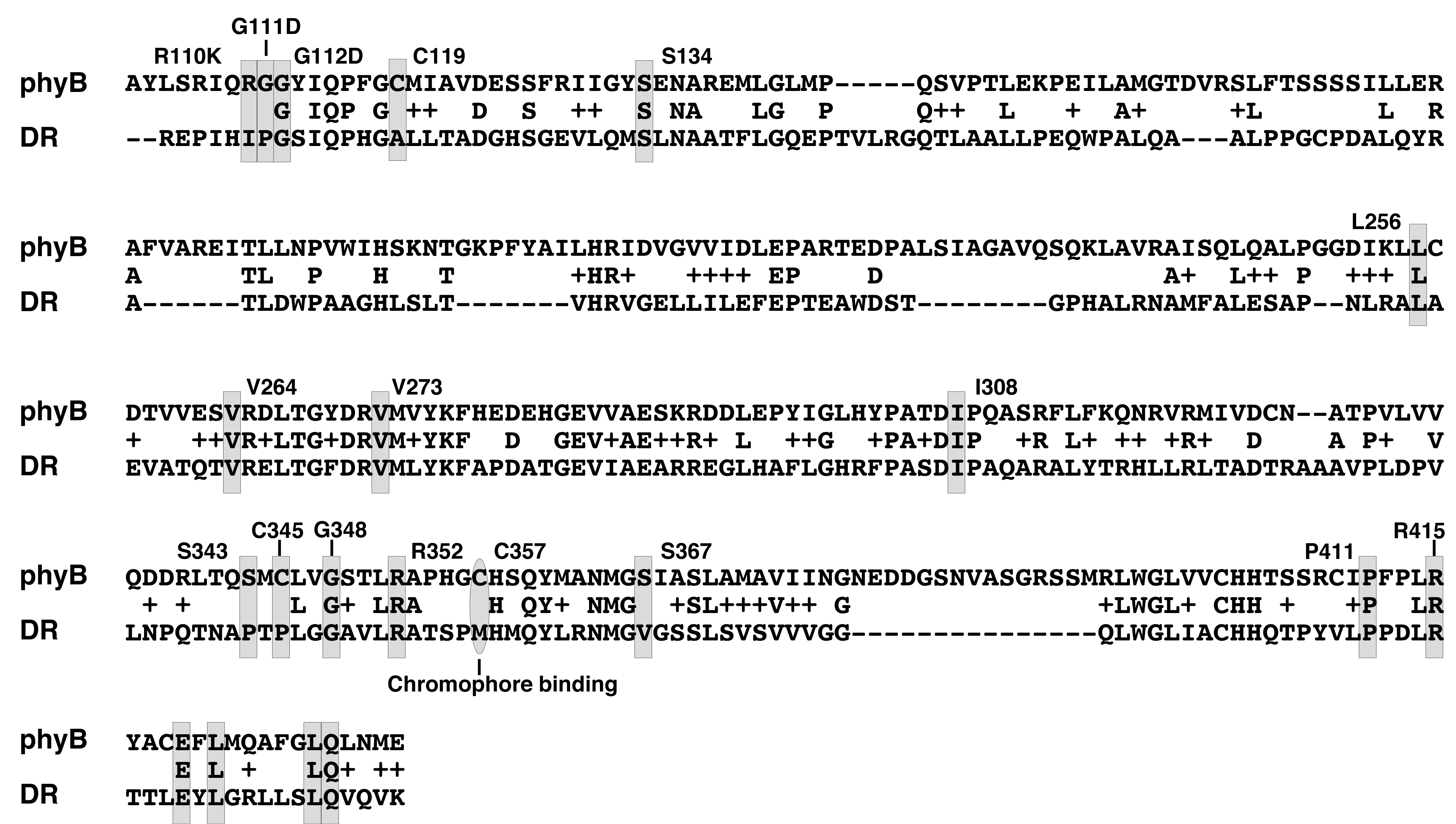

Supplement: Figure S3 — Arabidopsis phyB Missense Mutations Map Throughout the region of the Deinococcus radiodurans Phytochrome Used for Structure Determination. Pairwise sequence alignment between Arabidopsis phyB and phytochrome from the bacteria Deinococcus. phyB - Arabidopsis phyB; Dr - Deinococcus phytochrome; E-value - 1.16384E-28; 29% identical. Conserved residues are indicated. Boxes indicate positions of Arabidopsis phyB missense mutations that disrupt binding to PIF3. Oval indicates position of chromophore attachment in Arabidopsis. (0.41 MB TIF) [file pgen.1000352.s003.tif]

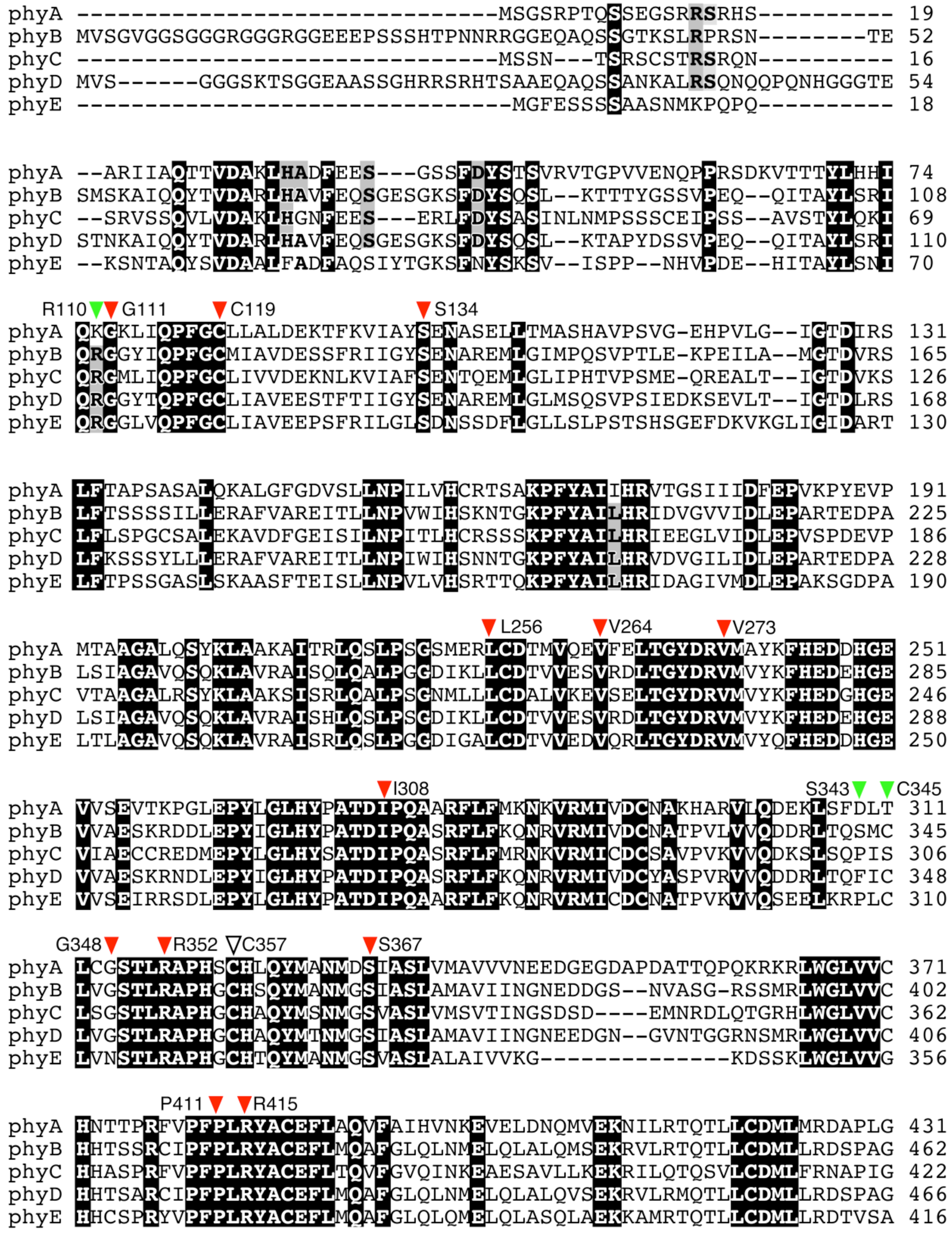

Supplement: Figure S4 — Multiple Sequence Alignment of Arabidopsis Phytochromes. Conserved residues are highlighted in reverse contrast. Arrows indicate amino acid residues defined here as being required for phyB binding to PIF3. Red arrows mark conserved residues, and green arrows mark non-conserved residues. (3.13 MB TIF) [file pgen.1000352.s004.tif]
